# Barriers to treated bednet usage in Timor-Leste: an explaratory study

**DOI:** 10.1186/1753-6561-5-S1-P16

**Published:** 2011-01-10

**Authors:** AA Lover, BA Sutton, AJ Asy

**Affiliations:** 1 Department of Epidemiology and Public Health, National University of Singapore, Singapore 117597; 2 Timor-Leste Asistencia Integradu Saúde (TAIS) (Timor-Leste Integrated Health Assistance), Timor-Leste

## 

Timor-Leste has some of the highest malaria rates in Asia- the WHO reports that 100% of the population is at year-round risk. A 2007 survey estimated that ITN usage (30 day) was only 28.8% in the under-5 population, and the MDG report also highlights several large disparities in ITN usage across the population- 69.6% urban and 45.5% rural; and 54% of males and only 46% of females, according to the Timor-Leste National Statistics Directorate (2007) and The Millennium Development Goals, Timor-Leste (2009). There have been many qualitative surveys about attitudes towards ITN usage in Sub-Saharan Africa, but far fewer from SE Asia [1].

To more fully understand the barriers to usage in Timor-Leste, a series of nine focus group discussions were organized in July, 2010. These discussions covered a range of peri-urban and rural areas, and were separated by sex, to allow exploration of intra-household decision making processes. A total of 53 women and 46 men participated, all of whom were heads of households or decision makers, and owned at least one bednet. A range of social, logistic and economic barriers emerged from these discussions, and could facilitate the creation of more targeted behavior-change materials.
